# Beyond Resection: Surgery as an Evolutionary Bottleneck Shaping Tumor Evolution and Treatment Response in Diffuse Gliomas

**DOI:** 10.3390/cancers18061012

**Published:** 2026-03-20

**Authors:** Paolo Tini, Flavio Donnini, Giovanni Rubino, Giuseppe Battaglia, Pierpaolo Pastina, Marta Vannini, Tommaso Carfagno, Giacomo Tiezzi, Ludovica Cellini, Giuseppe Minniti, Salvatore Chibbaro

**Affiliations:** 1 Unit of Radiation Oncology, Department of Medicine, Surgery and Neurosciences, University of Siena, 53100 Siena, Italy; flavio.donnini@student.unisi.it (F.D.); g.rubino@ao-siena.toscana.it (G.R.); giuseppe.battaglia@ao-siena.toscana.it (G.B.); pierpaolo.pastina@ao-siena.toscana.it (P.P.); m.vannini@ao-siena.toscana.ti (M.V.); t.carfagno@ao-siena.toscana.it (T.C.); 2 Unit of Neurosurgery, Department of Medicine, Surgery and Neurosciences, University of Siena, 53100 Siena, Italy; giacomo.tiezzi@ao-siena.toscana.it (G.T.); ludovica.cellini@ao-siena.toscana.it (L.C.); salvatore.chibbaro@unisi.it (S.C.); 3 Radiation Oncology, Policlinico Umberto I, Department of Radiological, Oncological and Pathological Sciences, “Sapienza” University of Rome, 00161 Rome, Italy; giuseppe.minniti@uniroma1.it; 4 IRCCS Neuromed, 86077 Pozzilli, Italy

**Keywords:** central nervous system tumors, diffuse glioma, glioblastoma, surgical resection, tumor microenvironment, clonal evolution, molecular margin, radiotherapy, treatment resistance, precision neuro-oncology

## Abstract

Surgical resection remains a cornerstone in the multidisciplinary management of central nervous system (CNS) tumors, particularly diffuse gliomas. Traditionally, the role of surgery has been evaluated primarily through quantitative metrics such as extent of resection and its association with survival outcomes. However, despite maximal and radiologically complete resections, recurrence remains nearly universal in malignant CNS tumors, suggesting that surgical cytoreduction alone does not fully account for post-surgical disease dynamics. Emerging biological and molecular evidence indicates that surgery represents not merely a technical intervention, but a biologically active event that profoundly reshapes tumor evolution and treatment response. In this review, we propose a conceptual framework that redefines surgery as a key biological driver in CNS tumor progression. We synthesize evidence demonstrating that surgical trauma induces inflammation, hypoxia, vascular remodeling, immune modulation, and extracellular matrix reorganization, collectively reprogramming the residual tumor microenvironment. These changes create selective pressures that favor the survival and expansion of adaptive tumor cell subpopulations, including invasive and stem-like phenotypes. From an evolutionary perspective, surgical resection functions as an acute selective bottleneck acting on heterogeneous tumor ecosystems, contributing to clonal selection and molecular divergence at recurrence. We further examine the dissociation between surgical (anatomical) margins and molecular (biological) margins, highlighting how biologically active tumor cells infiltrate beyond radiologically defined boundaries. This discrepancy provides a biological explanation for marginal and distant recurrences and challenges anatomy-based paradigms of surgical completeness. Importantly, we discuss how surgery-induced biological changes influence postoperative radiotherapy and systemic therapies, affecting radiosensitivity, target delineation, and therapeutic vulnerability. Finally, we outline future directions toward surgery-integrated precision neuro-oncology, emphasizing the potential of spatial profiling, liquid biopsy, advanced imaging, and artificial intelligence to capture perioperative tumor evolution. By reframing surgery as a biological inflection point rather than a neutral prelude to adjuvant treatment, this review advocates for a dynamic, biology-driven continuum of care aimed at anticipating tumor adaptation and improving long-term disease control in CNS tumors.

## Simple Summary

Surgery is a cornerstone in the treatment of central nervous system tumors, especially diffuse gliomas. Traditionally, its value has been measured mainly by how much tumor can be removed. However, most malignant CNS tumors recur even after apparently complete resections, indicating that surgery affects more than tumor volume. In this review, we discuss how surgical injury can reshape the tumor microenvironment by inducing inflammation, hypoxia, vascular and extracellular matrix remodeling, and immune changes. These postoperative conditions may favor adaptive tumor cell populations and influence where and how tumors recur, as well as how they respond to radiotherapy and systemic treatments. We propose that surgery should be considered a biological inflection point that contributes to tumor evolution. Recognizing this may help refine postoperative risk stratification and support more personalized, biology-informed adjuvant strategies.

## 1. Introduction

Surgical resection remains a central component of the multidisciplinary management of tumors of the central nervous system (CNS), particularly diffuse gliomas. Despite major advances in molecular classification, imaging, and adjuvant therapies, cytoreductive surgery continues to represent the first therapeutic intervention for most patients. In contemporary neuro-oncology, surgery is still primarily evaluated in terms of extent of resection (EOR) and its association with survival outcomes. Large modern series and meta-analyses have consistently confirmed that a greater EOR correlates with improved overall survival in glioblastoma and other diffuse gliomas, reinforcing the paradigm of “maximal safe resection” as a cornerstone of treatment [1,2]. However, accumulating clinical and biological evidence suggests that the relationship between surgical cytoreduction and outcome is more complex than a simple quantitative measure of tumor removal. Even in the context of gross total resection, the majority of malignant CNS tumors recur, often with aggressive behavior and limited responsiveness to subsequent therapies. Contemporary analyses have shown that survival curves frequently overlap across different degrees of resection, particularly when molecular features are taken into account, indicating that EOR alone does not fully capture post-surgical disease dynamics [3,4].

One major limitation of surgery lies in the biological diffusivity of CNS tumors. Modern imaging–pathology correlation studies have demonstrated that tumor cells with distinct molecular and transcriptional programs persist outside the resection cavity, even after apparently complete resections [5]. These findings challenge the notion that anatomical completeness equates to biological eradication.

In parallel, increasing attention has been devoted to the tumor microenvironment as a critical determinant of tumor behavior, recurrence, and treatment resistance. The CNS tumor microenvironment is now recognized as an active and adaptive ecosystem, composed of tumor cells, resident microglia, infiltrating macrophages, endothelial cells, and extracellular matrix components. Recent comprehensive reviews have highlighted the role of neuroinflammation, immune suppression, and hypoxia in sustaining tumor growth and mediating resistance to radiotherapy and systemic treatments [6,7,8,9].

Importantly, surgical intervention itself represents a profound biological perturbation of this ecosystem. Tissue injury induced by resection triggers inflammatory cascades, alters vascular permeability, disrupts the blood–brain barrier, and reshapes oxygen and nutrient gradients in the peritumoral region. Experimental and clinical data indicate that these surgery-induced changes can promote tumor cell plasticity, invasion, and resistance mechanisms, potentially influencing early recurrence patterns [10,11].

From an evolutionary standpoint, cancer progression is increasingly understood as a dynamic process driven by selective pressures acting on heterogeneous tumor cell populations. Within this framework, therapeutic interventions, including surgery, may function as evolutionary bottlenecks, eliminating sensitive clones while allowing resistant subpopulations to expand. Longitudinal genomic studies have provided compelling evidence that gliomas undergo marked clonal evolution between diagnosis and recurrence. Using paired primary and recurrent tumor samples, Barthel et al. [12] and Kim et al. [13] demonstrated that recurrent tumors frequently harbor distinct genetic and transcriptional profiles, consistent with treatment-driven selection.

The clinical relevance of tumor evolution has been further underscored by the integration of molecular profiling into the World Health Organization (WHO) classification of CNS tumors. Large-scale studies have shown that molecular features such as IDH mutation status, TERT promoter alterations, EGFR amplification, and H3 histone mutations not only define prognosis but also influence patterns of invasion, recurrence, and therapeutic vulnerability [14,15]. Within this context, it remains uncertain whether surgical strategies grounded exclusively in anatomical criteria are sufficient to account for the biological heterogeneity and infiltrative behavior of CNS tumors.

Collectively, these observations support a shift in perspective: surgery should no longer be regarded merely as a technical step preceding adjuvant therapies, but rather as a biological driver that actively shapes tumor evolution and treatment response. Understanding how surgical trauma modulates the tumor microenvironment, exerts selective pressure on residual tumor cells, and interacts with radiotherapy and systemic treatments is essential for refining postoperative strategies. In this review, we explore the emerging concept of surgical biology in CNS tumors, examining its implications for recurrence patterns, molecular evolution, and the development of integrated, biology-driven approaches to precision neuro-oncology.

## 2. Surgical Trauma as a Biological Event

**Figure 1:**
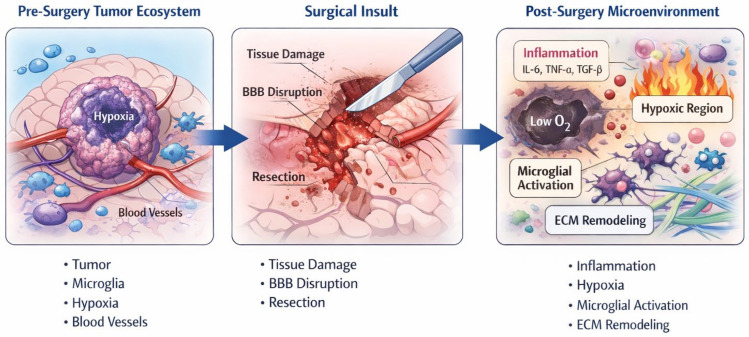
Surgery as a biological event reshaping the tumor microenvironment. Schematic representation of the biological consequences of surgical resection in CNS tumors. The pre-surgical tumor ecosystem is characterized by heterogeneous tumor cell populations, abnormal vasculature, baseline hypoxia, and a dynamic interaction with resident immune and stromal cells. Surgical resection represents an acute biological insult, inducing tissue injury, vascular disruption, and breakdown of the blood–brain barrier. In the post-surgical setting, inflammatory signaling, immune cell activation, peri-cavitary hypoxia, and extracellular matrix remodeling collectively reshape the residual tumor niche. These surgery-induced changes may promote adaptive tumor phenotypes and influence recurrence patterns and response to adjuvant therapies.

Surgical resection of CNS tumors has traditionally been conceptualized as a mechanical intervention aimed at cytoreduction and symptom relief. However, accumulating experimental and clinical evidence indicates that surgery constitutes a profound biological perturbation of the tumor ecosystem, with consequences that extend well beyond the immediate removal of tumor tissue. Rather than representing a neutral reset of the disease burden, surgical trauma initiates a cascade of inflammatory, vascular, and metabolic changes that reshape the residual tumor microenvironment and may influence subsequent tumor behavior (Figure 1).

Tissue injury induced by resection triggers an acute inflammatory response characterized by the release of cytokines, chemokines, and growth factors. Recent studies have shown that surgical trauma promotes the activation of microglia and recruitment of peripheral macrophages, leading to a transient but biologically relevant pro-inflammatory milieu within the peritumoral brain. Buonfiglioli et al. [16] reported that microglia–macrophage activation states are highly plastic and can shift toward tumor-supportive phenotypes in response to tissue damage. Similarly, Van der Sanden et al. reported that post-surgical inflammatory signaling may enhance tumor cell survival and invasiveness through cytokine-mediated pathways [17].

In parallel, surgical disruption of the blood–brain barrier (BBB) represents a critical biological consequence of tumor resection. BBB breakdown alters vascular permeability, facilitates immune cell infiltration, and modifies the pharmacokinetics of adjuvant therapies. Advanced imaging and molecular studies have shown that BBB permeability remains altered for weeks following surgery, potentially creating spatially heterogeneous niches within the resection cavity and surrounding brain tissue. Brooks and Parrinello highlighted how vascular and endothelial responses to tissue injury can contribute to adaptive tumor responses, including angiogenesis and metabolic reprogramming [18].

Another key consequence of surgical trauma is the generation of peri-cavitary hypoxia. Resection inevitably disrupts local blood supply, leading to transient ischemia and altered oxygen gradients in the surrounding brain parenchyma. Hypoxic conditions are well recognized as potent drivers of tumor aggressiveness, promoting stemness, invasion, and resistance to radiotherapy. Recent translational studies have demonstrated that hypoxia-inducible signaling pathways, including HIF-1α-mediated transcriptional programs, are upregulated in residual tumor cells located near the resection margin. Singh et al. and Bayona et al. provided evidence that hypoxic stress fosters adaptive phenotypes associated with treatment resistance and recurrence [8,19].

Beyond inflammatory and hypoxic effects, surgical trauma induces extensive remodeling of the extracellular matrix (ECM) and mechanical properties of the tumor niches. Changes in ECM composition and stiffness have been shown to modulate tumor cell migration and invasion. Suarez-Meade et al. emphasized that physical and biochemical alterations of the tumor microenvironment following therapeutic interventions can actively shape tumor evolution [20]. In the post-surgical setting, ECM remodeling may facilitate the dispersal of invasive tumor cells into surrounding brain tissue, contributing to diffuse recurrence patterns.

Collectively, these processes support the notion that surgical resection initiates a biological reprogramming of the tumor microenvironment rather than simply debulking disease. The biological consequences of surgery may also vary according to the extent of resection (EOR). Supratotal resection, gross total resection, subtotal resection, and biopsy represent markedly different biological scenarios in terms of residual tumor burden and spatial distribution of surviving tumor cells. In the setting of supratotal resection, the peri-cavitary microenvironment may contain relatively fewer tumor cells but may still harbor infiltrative populations along white matter tracts. In contrast, subtotal resection or biopsy leaves a larger residual tumor mass that may maintain pre-existing tumor–microenvironment interactions while simultaneously experiencing surgery-induced inflammatory and hypoxic stress. These differences suggest that the postoperative tumor niche is not uniform but varies according to surgical strategy, potentially influencing patterns of recurrence and response to adjuvant therapies [3,21].

As schematically illustrated in Figure 1, the transition from the pre-surgical tumor ecosystem to the post-surgical microenvironment involves coordinated changes in inflammation, vascular integrity, oxygenation, and stromal interactions. These surgery-induced alterations may create selective conditions that favor the survival and expansion of tumor cell subpopulations with enhanced adaptive capacity, thereby influencing early recurrence dynamics and response to adjuvant therapies.

Recognizing surgical trauma as a biologically active event has important implications for postoperative management. It suggests that the timing, targeting, and intensity of adjuvant radiotherapy and systemic treatments should be considered in light of the evolving post-surgical tumor ecosystem. Rather than treating surgery and adjuvant therapies as sequential and independent steps, a biologically informed integration of surgical and postoperative strategies may be required to counteract surgery-induced adaptive responses.

## 3. Clonal Selection and Tumor Evolution After Surgical Resection

Diffuse CNS tumors, and gliomas in particular, are characterized by marked intratumoral heterogeneity at genetic, epigenetic, and transcriptional levels [22]. Contemporary molecular profiling studies have demonstrated that even treatment-naïve tumors harbor multiple coexisting subclones with distinct biological properties, including differential proliferative capacity, invasive potential, and therapeutic sensitivity [23]. This intrinsic heterogeneity provides the substrate upon which therapeutic interventions, including surgery, can act as selective pressures, shaping tumor evolution over time.

From an evolutionary perspective, surgical resection can be conceptualized as an acute selective bottleneck that dramatically reduces tumor burden while simultaneously altering the ecological context in which residual tumor cells persist. Rather than eliminating the disease uniformly, surgery preferentially removes spatially accessible and often more proliferative tumor populations, while leaving behind subclones adapted to hostile microenvironmental conditions such as hypoxia, nutrient deprivation, and immune surveillance. This concept aligns with modern evolutionary models of cancer progression, in which treatment-induced bottlenecks favor the expansion of resistant phenotypes rather than complete eradication [24].

Compelling evidence for surgery-driven clonal selection has emerged from longitudinal genomic analyses of paired primary and recurrent CNS tumors. Using integrative genomic and transcriptomic profiling, Barthel et al. [12] demonstrated that recurrent gliomas frequently diverge substantially from their primary counterparts, acquiring novel driver alterations and transcriptional programs consistent with adaptive evolution under therapeutic pressure. Importantly, these changes were not random but followed reproducible evolutionary trajectories, suggesting that treatment-related selective forces actively shape recurrence biology.

Spatially resolved analyses have further refined this concept by revealing that distinct tumor regions contribute differentially to recurrence [25]. Jung et al. [26] highlighted that subclones residing at the invasive margins of the tumor, often less affected by surgical resection, are disproportionately represented in recurrent disease. These marginal populations frequently exhibit gene expression signatures associated with migration, stemness, and therapy resistance, supporting the hypothesis that surgery selects for biologically aggressive phenotypes rather than merely reducing tumor volume [27].

Tumor stem-like cells appear to play a central role in this process. Glioma stem cells (GSCs), characterized by enhanced self-renewal capacity and resistance to cytotoxic therapies, are preferentially enriched in hypoxic and peri-vascular niches that may be relatively preserved following resection [28]. Recent studies have shown that surgical trauma and subsequent microenvironmental remodeling can further promote stem-like states through inflammatory and hypoxia-driven signaling pathways providing mechanistic evidence linking microenvironmental stress to stem cell enrichment and clonal dominance at recurrence [29,30]. Importantly, clonal selection after surgery is not limited to genetic alterations but also involves epigenetic and transcriptional reprogramming. Single-cell sequencing studies have revealed that residual tumor cells can undergo rapid phenotypic transitions in response to post-surgical cues, adopting mesenchymal or invasive transcriptional states associated with poor prognosis [31]. Neftel et al. [32] demonstrated that glioblastoma cells dynamically transition between cellular states rather than remaining fixed in discrete lineages, highlighting the plastic nature of tumor evolution. Surgical perturbation may accelerate these transitions by reshaping the surrounding niche.

**Figure 2:**
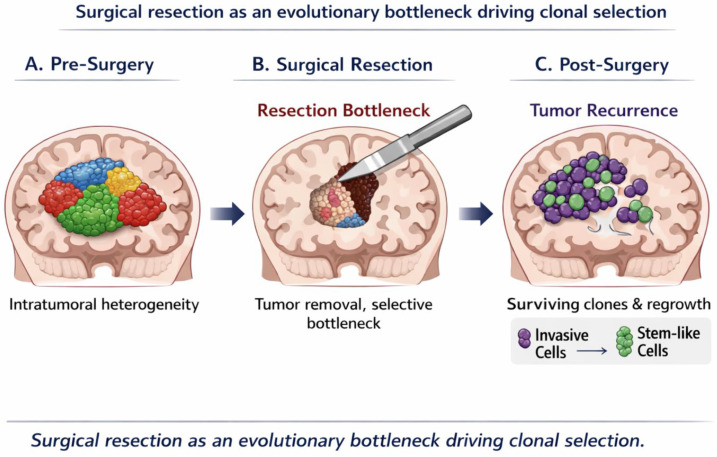
Surgical resection as an evolutionary bottleneck driving clonal selection. This evolutionary framework highlights how surgery can shape tumor biology beyond its cytoreductive effect. Conceptual model illustrating how surgical intervention may act as an evolutionary bottleneck in diffuse gliomas. (**A**) Before surgery, the tumor contains multiple coexisting subclones with distinct biological properties and spatial niches. (**B**) Surgical resection removes a large proportion of tumor cells but imposes a strong selective pressure on the tumor ecosystem. (**C**) Residual tumor populations with enhanced adaptive capacity—such as invasive or stem-like phenotypes—survive and expand, ultimately driving tumor recurrence and progression. This model highlights how surgery may shape tumor evolution beyond its cytoreductive effect.

Collectively these findings support a model in which surgical resection acts as an evolutionary filter, selectively favoring tumor cell populations with enhanced adaptive capacity (Figure 2). The biological properties of the residual disease, rather than the absolute extent of resection, may therefore be critical determinants of recurrence pattern, aggressiveness, and response to adjuvant therapies. Recognizing surgery as a driver of clonal selection provides a conceptual framework for understanding why anatomically complete resections do not necessarily translate into durable disease control and underscores the need for postoperative strategies tailored to the evolving biology of residual tumor cells.

## 4. Surgical Margins Versus Molecular Margins

The concept of surgical margins has historically been grounded in anatomical and radiological criteria, with gross total resection commonly defined by the absence of contrast-enhancing tumor on postoperative magnetic resonance imaging [33]. While this approach remains clinically relevant, growing molecular and biological evidence indicates that anatomical margins do not coincide with the true biological extent of CNS tumors. In diffuse gliomas and other infiltrative brain tumors, residual disease often persists beyond visible surgical boundaries, challenging the adequacy of anatomy-based definitions of completeness.

Recent molecular and imaging studies have demonstrated that tumor cell infiltration extends far beyond radiographically detectable lesions [34]. Using advanced imaging-genomic correlations, Gill et al. [21] showed that tumor-associated molecular alterations can be detected in non-enhancing brain regions adjacent to the surgical cavity. These findings support the notion that the apparent surgical margin frequently underestimates the spatial distribution of biologically active tumor cells.

At the molecular level, residual tumor cells located at the invasive front often harbor distinct genetic and transcriptional profiles compared with cells from the tumor core. Bhaduri et al. [35] demonstrated that peripheral tumor populations exhibit stem-like and migratory phenotypes associated with treatment resistance. Similarly, Nomura et al. [36] identified transcriptional programs at the tumor–brain interface that promote invasion and adaptation, underscoring the biological relevance of marginal disease.

The dissociation between surgical and molecular margins is further amplified by intratumoral heterogeneity and clonal evolution. As discussed in the previous section, surgical resection acts as an evolutionary bottleneck, preferentially removing central tumor populations while sparing infiltrative cells at the periphery. These marginal cells frequently exhibit molecular features associated with aggressive behavior, including mesenchymal transition, metabolic flexibility, and resistance to radiotherapy and chemotherapy [32]. Suvà and Tirosh [37] highlighted how spatially distinct tumor compartments contribute disproportionately to recurrence, emphasizing that the biological “edge” of the tumor may be more relevant than the radiological boundary.

Molecular classification has further refined this concept. In IDH-wildtype glioblastoma, alterations such as EGFR amplification, TERT promoter mutations, and chromosomal instability have been associated with highly infiltrative growth patterns that extend beyond surgical margins. Conversely, IDH-mutant gliomas often demonstrate diffuse infiltration along white matter tracts despite relatively indolent radiological appearances. Large integrative analyses by Louis et al. [14] and Weller et al. [38] underscore that molecular features, rather than anatomical extent alone, are key determinants of tumor behavior and recurrence risk.

These observations have profound implications for postoperative management, particularly for radiotherapy planning. Target volume delineation based solely on surgical cavities and contrast-enhancing disease may fail to encompass biologically relevant residual tumor regions [39]. Emerging evidence suggests that incorporating molecular and functional imaging biomarkers, such as diffusion abnormalities, metabolic signatures, or radiomic features, may better reflect the true extent of disease. Kickingereder et al. [40] demonstrated that radiomic features beyond contrast enhancement correlate with molecular aggressiveness and recurrence patterns, supporting a shift toward biologically informed target definition.

**Figure 3:**
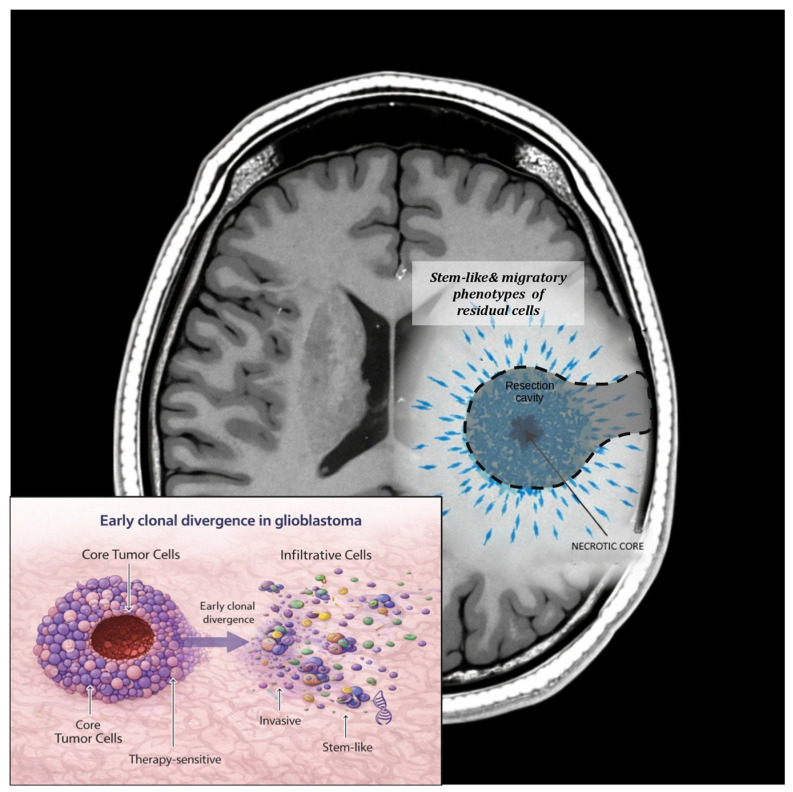
Surgical margins versus molecular margins in CNS tumors.

As illustrated in Figure 3, the discrepancy between surgical and molecular margins represents a fundamental limitation of anatomy-based treatment paradigms. Recognizing that “complete resection” does not equate to biological eradication is essential for understanding recurrence dynamics and optimizing adjuvant therapies. A paradigm that integrates surgical anatomy with molecular and microenvironmental information may offer a more accurate framework for risk stratification and personalized postoperative treatment strategies.

Schematic representation of the discrepancy between anatomical and biological tumor boundaries. The surgical margin, defined by postoperative imaging and extent of resection, delineates the visible tumor cavity.

However, biologically active tumor cells may infiltrate beyond radiological limits into surrounding brain tissue. These infiltrative populations—often enriched in invasive or stem-like phenotypes—constitute the molecular margin of the tumor and may contribute to local, marginal, or distant recurrence.

## 5. Surgery, Recurrence Patterns, and Molecular Shift

Despite maximal surgical resection and contemporary multimodal treatment, recurrence remains an almost universal feature of malignant CNS tumors, particularly glioblastoma. Importantly, tumor recurrence is not a random event but follows reproducible spatial and biological patterns that reflect the interaction between surgical intervention and tumor biology. Increasing evidence suggests that surgery not only influences the location of recurrence but also shapes its molecular and phenotypic characteristics, contributing to a progressive shift toward more aggressive disease.

Several modern imaging-based studies have demonstrated that the majority of glioblastoma recurrences arise within or adjacent to the surgical cavity, yet a substantial proportion occur at the margins or at distant sites, particularly in tumors with highly infiltrative growth patterns. Using longitudinal imaging analyses, Chiranth et al. [41] reported that distant progression is an aggressive progression pattern associated with poor prognosis. These findings indicate that recurrence patterns are not solely dictated by treatment fields but are deeply influenced by the biological properties of residual tumor cells.

From a molecular standpoint, recurrent tumors often differ significantly from their primary counterparts. Integrated genomic studies have shown that surgical resection and subsequent therapies select for tumor cell populations with distinct genetic and transcriptional profiles. Barthel et al. [12] demonstrated that recurrent gliomas frequently acquire novel driver alterations and chromosomal aberrations that were absent or subclonal at diagnosis. This molecular divergence supports the concept that recurrence represents a selective evolved disease state rather than simple regrowth of the primary tumor.

Single-cell and bulk RNA sequencing studies have revealed that recurrent glioblastomas are enriched for mesenchymal-like and stem-like transcriptional states associated with invasiveness, immune evasion, and resistance to therapy. Neftel et al. [32] showed that glioblastoma cells dynamically transition between cellular states, with therapy acting as a catalyst for phenotypic switching. Surgical trauma, adjuvant therapies and the associated microenvironmental changes described in Section 2 may initiate and accelerate this plasticity, favoring the emergence of aggressive recurrent phenotypes.

Spatial analyses have further linked molecular evolution to recurrence patterns. Tumor cells located at the invasive front, often beyond the surgical margin, are more likely to seed marginal or distant recurrences and exhibit molecular features distinct from those of the tumor core. Bhaduri et al. [35] identified peripheral tumor populations with enhanced migratory capacity and resistance to cytotoxic stress, underscoring the biological relevance of marginal disease. These observations align with the concept of a molecular margin extending beyond the anatomical resection boundary, as discussed in Section 4.

The molecular shift observed at recurrence has important clinical implications. Recurrent tumors frequently display reduced sensitivity to standard therapies, including radiotherapy and temozolomide, and may exhibit altered immune landscapes that limit the efficacy of immunotherapeutic approaches. Wang et al. [42] reported significant changes in immune cell composition and checkpoint expression between primary and recurrent tumors, highlighting the adaptive nature of the disease. These changes may, in part, reflect surgery-induced spatial and biological selection pressures acting on heterogeneous tumor populations.

**Figure 4:**
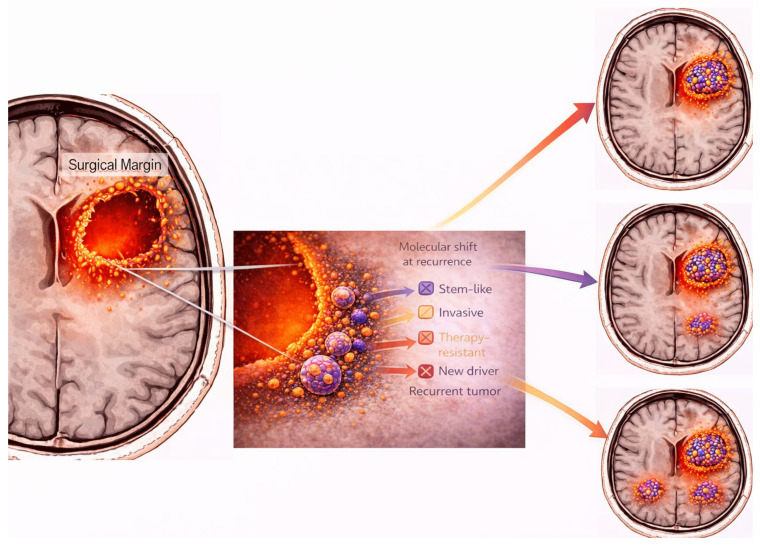
Biological mechanisms underlying glioblastoma recurrence after surgical resection. Conceptual overview of the processes contributing to tumor recurrence after surgery. Residual molecularly active tumor cells located beyond the surgical margin may survive treatment and drive disease progression. Selective pressures imposed by surgery and subsequent therapies promote clonal evolution, leading to molecular and phenotypic shifts at recurrence, including increased invasiveness, stem-like features, therapy resistance, and acquisition of new oncogenic drivers.

Collectively, these findings support a model in which surgery influences not only when and where tumors recur, but also how they recur at the molecular level. Recurrence should therefore be viewed as the outcome of an evolutionary process shaped by surgical cytoreduction, microenvironmental remodeling, and subsequent adjuvant therapies. As schematically summarized in Figure 4, the interplay between surgical margins, residual biologically active disease, and clonal evolution determines recurrence patterns and molecular progression.

Understanding the biological underpinnings of recurrence patterns may help refine postoperative risk stratification and guide more personalized treatment strategies. Rather than assuming biological continuity between primary and recurrent disease, clinicians and researchers should consider recurrence as a distinct, evolved entity that emerges from selective pressures imposed by surgery and multimodal therapy.

## 6. Implications for Radiotherapy and Adjuvant Therapies

**Figure 5:**
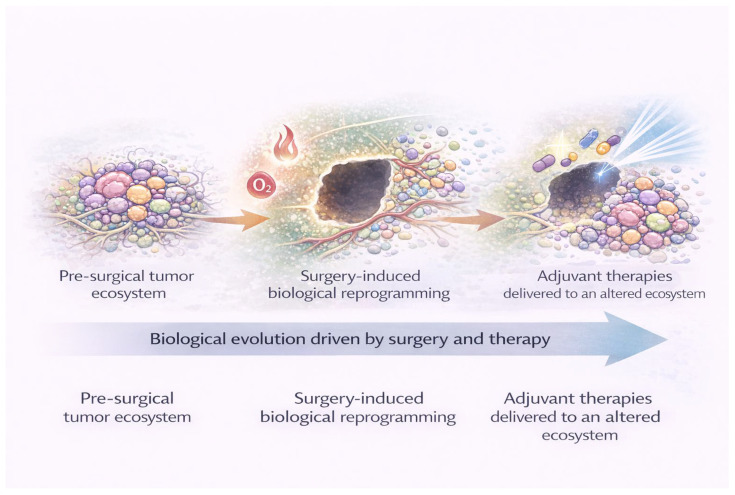
Surgery-informed biological context of adjuvant therapies in diffusive gliomas. Schematic representation of how surgical resection reshapes the tumor microenvironment and influences the biological substrate on which adjuvant radiotherapy and systemic therapies are delivered. Surgery induces inflammation, hypoxia, vascular remodeling, and clonal selection, resulting in a biologically reprogrammed residual disease. Consequently, postoperative therapies act on an evolved tumor ecosystem rather than on the pre-surgical tumor, with important implications for treatment response and recurrence patterns.

The recognition of surgery as a biologically active event, rather than a purely cytoreductive intervention, has profound implications for postoperative radiotherapy and systemic treatments in CNS tumors. Traditional adjuvant strategies have largely been designed under the assumption that surgery merely reduces tumor burden, often underestimating the extent to which surgical intervention may alter tumor biology both spatially and molecularly. However, the evidence discussed in the preceding sections indicates that surgical resection reshapes the tumor microenvironment, drives clonal selection, and contributes to molecular evolution, thereby influencing treatment response and patterns of failure as schematically illustrated in Figure 5. Radiotherapy remains a cornerstone of postoperative management in high-grade gliomas, yet its efficacy is tightly linked to the biological characteristics of residual disease. Surgery-induced hypoxia, inflammation, and vascular remodeling can directly modulate radiosensitivity. Hypoxic tumor cells are well known to exhibit reduced responsiveness to ionizing radiation due to impaired oxygen-mediated fixation of DNA damage. Recent translational studies have demonstrated that peri-cavitary hypoxia following surgical resection may persist during the early postoperative period, potentially reducing the effectiveness of adjuvant radiotherapy when delivered to biologically unfavorable niches. Horsman and Overgaard [43] emphasized that spatial and temporal hypoxia remains a critical determinant of radiation response in brain tumors.

Target volume delineation represents another major challenge. Contemporary radiotherapy planning is primarily guided by anatomical imaging, incorporating the surgical cavity and surrounding margins based on contrast enhancement and edema. However, as illustrated above, the molecular margin of disease often extends beyond these radiological boundaries. Failure to account for biologically active infiltrative tumor cells may contribute to a high rate of recurrences. Advanced imaging and radiomic approaches have shown promise in identifying regions of aggressive biology beyond contrast-enhancing disease. Kim et al. [44] demonstrated that radiomic features derived from non-enhancing regions correlate with molecular aggressiveness and recurrence risk, supporting a shift toward biologically informed target definition.

Surgical biology also intersects with systemic therapy responsiveness. Temozolomide efficacy, for instance, may be influenced by the selective survival of resistant subclones enriched at the tumor margins following resection. Molecular evolution at recurrence frequently includes alterations associated with DNA repair, cell cycle regulation, and metabolic adaptation, which may reduce sensitivity to alkylating agents. Barthel et al. [12] and Kim et al. [13] provided evidence that recurrent tumors represent biologically distinct entities with altered therapeutic vulnerabilities.

Immunotherapeutic strategies are similarly affected by surgery-induced changes in the tumor microenvironment. Surgical trauma triggers inflammatory signaling and immune cell recruitment, yet this response may paradoxically promote immunosuppressive phenotypes within the tumor niche. Recent studies have highlighted dynamic shifts in immune cell composition and checkpoint expression following standard therapy, including surgery. Pombo Antunes et al. [45] showed that macrophage polarization and T-cell dysfunction evolve over time, potentially limiting the efficacy of immune-based treatments if not appropriately timed or combined with other modalities.

These observations collectively argue for a more integrated approach to postoperative therapy that accounts for the evolving biological landscape shaped by surgery. Rather than viewing surgery, radiotherapy, and systemic treatments as sequential and independent steps, a surgery-informed, biology-driven therapeutic framework may be required. This could include adaptive radiotherapy strategies guided by molecular imaging, optimized timing of adjuvant treatments to counteract surgery-induced hypoxia or inflammation, and tailored systemic therapies targeting biologically selected residual disease.

In this context, surgery should be considered an integral component of precision neuro-oncology, not only as a means of tissue removal but also as a source of biological information and a determinant of subsequent treatment response. Incorporating surgical biology into postoperative decision-making may help refine risk stratification, improve local control, and ultimately mitigate the aggressive evolutionary trajectories that characterize recurrent CNS tumors.

## 7. Future Directions: Toward Surgery-Integrated Precision Neuro-Oncology

The cumulative evidence reviewed in the preceding sections supports a paradigm shift in neuro-oncology, suggesting that surgery may act as an evolutionary bottleneck shaping tumor biology. Rather than representing a purely cytoreductive step preceding adjuvant therapies, surgical resection emerges as a central biological determinant of tumor evolution, recurrence patterns, and treatment response. This recognition opens new avenues for integrating surgical information into precision neuro-oncology frameworks.

One major future direction lies in the biological exploitation of surgical intervention. Surgical specimens already serve as the primary source for histological and molecular diagnosis; however, their potential to inform postoperative risk stratification remains underutilized. Advances in spatial transcriptomics and single-cell sequencing now allow high-resolution mapping of tumor heterogeneity and microenvironmental interactions across different tumor regions, including the invasive margin. Ravi et al. [46] demonstrated that molecular programs associated with invasion and therapy resistance are spatially enriched at tumor–brain interfaces, suggesting that surgical margin-specific profiling could provide actionable biological insights.

Beyond tissue-based analyses, surgery may also act as a temporal anchor for dynamic disease monitoring. Liquid biopsy approaches, including circulating tumor DNA and extracellular vesicles, have shown promise in capturing tumor evolution longitudinally. Miller et al. [47] and Sabedot et al. [48] highlighted how postoperative molecular dynamics may reflect residual disease burden and emerging resistance mechanisms. Future translational studies will be essential to experimentally validate the hypothesis that surgical intervention actively shapes tumor evolution. Several complementary strategies could be envisioned. First, spatial multi-omics analyses of tumor samples collected from the resection cavity margins may help characterize perioperative microenvironmental changes and identify surgery-induced transcriptional programs [46]. Second, in vitro models exposing glioma cells to inflammatory cytokines, hypoxia, or extracellular matrix remodeling signals associated with surgical injury could clarify how these factors influence tumor plasticity and therapeutic resistance [8,19,20]. Third, in vivo experimental models comparing tumor evolution after different extents of surgical resection may provide mechanistic insight into the evolutionary bottleneck imposed by surgery, in line with current evolutionary frameworks of cancer progression [24]. Finally, perioperative liquid biopsy approaches may allow real-time monitoring of clonal dynamics following surgical intervention [47,48].

Artificial intelligence (AI) and advanced imaging analytics represent another critical frontier. Radiomic and deep learning approaches applied to perioperative imaging have demonstrated the ability to infer molecular features, invasive behavior, and recurrence risk from standard MRI sequences. Kickingereder et al. [49] and Chang et al. [50] suggested that AI-derived imaging biomarkers could bridge the gap between anatomical margins and molecular disease, enabling surgery-informed radiotherapy planning and adaptive treatment strategies.

These technological advances also raise the possibility of adaptive, biology-guided adjuvant therapy. Rather than applying uniform postoperative treatment protocols, future strategies may tailor radiotherapy volumes, dose distributions, and systemic therapy choices based on surgery-induced biological states. Early-phase studies exploring adaptive radiotherapy and response-guided treatment intensification provide proof-of-concept that dynamic treatment modification is feasible. Tseng et al. [51] emphasized that integrating biological information into adaptive workflows may improve local control while limiting unnecessary toxicity. These emerging biological insights may also open the possibility of adapting neurosurgical strategies to mitigate surgery-induced tumor-promoting signals. Potential approaches include supratotal resection when functionally feasible, intraoperative technologies for detecting infiltrative tumor margins, local delivery of anti-tumor agents within the surgical cavity, and integration of intraoperative molecular sampling. Although these strategies remain largely investigational, they highlight how neurosurgical practice could evolve within a biology-informed therapeutic framework, building on evidence linking extent of resection, residual disease biology, and injury-induced tumor adaptation [3,18,21].

Crucially, realizing a surgery-integrated precision oncology model will require a shift from discipline-specific workflows to truly multidisciplinary integration. Surgeons, radiation oncologists, medical oncologists, molecular pathologists, and data scientists must collaborate around a shared biological framework, in which surgical decisions are informed by anticipated downstream effects on tumor evolution and therapeutic response. Such integration aligns with emerging systems-level approaches to cancer care, as advocated by Prados et al. [52].

In this context, surgery should be repositioned not as the beginning of a linear treatment sequence, but as a biological inflection point that shapes the trajectory of disease. Embracing this perspective may facilitate the development of postoperative strategies that anticipate, rather than react to, tumor evolution. Ultimately, surgery-integrated precision neuro-oncology has the potential to transform postoperative management from a standardized protocol into a biologically informed, adaptive continuum of care.

## 8. Discussion: Surgery as a Biological Driver of Tumor Evolution in CNS Tumors

This review advances a conceptual reinterpretation of surgery in CNS tumors, proposing that surgical resection should be understood not merely as a cytoreductive intervention, but as a biologically active event that shapes tumor evolution, recurrence patterns, and treatment response. By integrating evidence from tumor biology, clonal evolution, imaging, and clinical outcomes, we argue that surgery represents a critical inflection point in the disease trajectory rather than a neutral prelude to adjuvant therapy. Importantly, recognizing surgery as a biologically transformative event opens the possibility of reconsidering how subsequent therapies are sequenced and integrated, with the goal of adapting treatment strategies to the biological state imposed by surgical intervention itself.

A central message emerging from this review is that anatomical completeness does not equate to biological completeness. While extent of resection remains an important prognostic factor, its clinical value cannot be fully understood without considering the biological properties of residual disease. The dissociation between surgical margins and molecular margins highlights a fundamental limitation of anatomy-based paradigms and provides a biological explanation for the high rate of recurrence observed even after radiologically complete resections. This perspective reconciles seemingly paradoxical observations in the literature, such as improved survival with greater resection without corresponding durable disease control. More importantly, it suggests that postoperative strategies should not simply assume a continuation of preoperative tumor biology, but rather be recalibrated in light of the new biological equilibrium established after surgery.

By framing surgery as an evolutionary bottleneck, this review situates surgical intervention within contemporary models of cancer evolution. Surgical resection imposes a strong selective pressure on heterogeneous tumor populations, preferentially eliminating dominant but therapy-sensitive clones while allowing adaptive, invasive, or stem-like populations to persist. The recurrent tumor that emerges is therefore not simply a regrowth of the primary lesion, but an evolved biological entity, shaped by the combined pressures of surgery and adjuvant therapies. This evolutionary framing provides a unifying explanation for molecular divergence at recurrence and for the frequent emergence of treatment resistance. It also implies that postoperative treatments might achieve greater efficacy if they are timed and selected to specifically target the biological characteristics of the residual, surgery-selected populations, rather than following rigid treatment sequences.

Importantly, this review emphasizes that the biological consequences of surgery extend beyond tumor cell selection to include microenvironmental reprogramming.

Evidence from other cancer types further supports the concept that surgical trauma may influence tumor biology. In breast and colorectal cancer, postoperative inflammatory responses and wound-healing signals have been associated with stimulation of residual tumor cells and metastatic growth. Experimental studies have shown that surgical wound fluids can promote tumor cell proliferation and invasion through cytokine-mediated signaling pathways [53], while systemic inflammatory responses to surgery may facilitate the outgrowth of previously controlled tumor deposits [54]. Although the biological context of brain tumors is distinct, these observations reinforce the broader oncologic principle that surgical injury may act as a biological modulator of tumor progression.

Surgical trauma induces inflammation, hypoxia, vascular remodeling, and immune modulation, all of which influence tumor cell behavior and therapeutic sensitivity. These changes challenge the implicit assumption that postoperative radiotherapy and systemic treatments act on a biological substrate comparable to the pre-surgical tumor. Instead, adjuvant therapies are delivered into a dynamically altered ecosystem, with important implications for treatment timing, target definition, and therapeutic efficacy. A deeper understanding of these processes may allow clinicians to integrate treatments that counteract or exploit surgery-induced biological changes, potentially improving local control and delaying resistance.

Another key contribution of this review lies in its multidisciplinary integration. By bridging neurosurgery, radiation oncology, medical oncology, molecular pathology, and imaging sciences, we propose a framework in which surgical decisions are informed not only by immediate anatomical considerations but also by anticipated downstream biological effects. Such a framework naturally leads to reconsideration of conventional treatment sequencing, encouraging closer coordination among specialties to design treatment pathways that dynamically respond to biology modified by prior interventions. This integrative perspective aligns with emerging precision oncology paradigms and underscores the need for closer collaboration across disciplines to address the complex biology of CNS tumors.

At the same time, several limitations should be acknowledged. Much of the evidence supporting surgery-induced biological effects is derived from retrospective analyses, preclinical models, or indirect clinical observations. Prospective studies specifically designed to capture perioperative biological dynamics remain scarce. Furthermore, translating biological insights into actionable changes in clinical practice, such as adaptive radiotherapy, surgery-informed systemic therapy, or modified treatment sequencing, will require careful validation to avoid overtreatment or unnecessary toxicity. Nonetheless, the convergence of evidence across multiple domains supports the validity and clinical relevance of the proposed conceptual framework.

In summary, the message of this review is not that surgery is detrimental, but that surgery matters biologically. Recognizing surgery as a driver of tumor evolution rather than a neutral intervention offers a more coherent understanding of recurrence, resistance, and treatment failure in CNS tumors. At the same time, acknowledging the biological impact of surgical resection invites a shift from rigid, linear treatment sequences toward adaptive therapeutic strategies that integrate and respond to treatment-induced biological changes. Ultimately, this perspective supports the development of a dynamic, biology-driven continuum of care, in which surgical intervention becomes a fully integrated component of precision neuro-oncology aimed at improving long-term patient outcomes.

## 9. Conclusions

Surgery should be viewed as a biological “inflection point” capable of shaping residual disease behavior and subsequent treatment response. Because anatomical completeness does not necessarily equate to biological completeness, the concept of a molecular margin provides a plausible framework to explain marginal and distant recurrences. Importantly, postoperative radiotherapy and systemic therapies are delivered into a dynamically altered ecosystem, reinforcing the need for biology-informed timing and targeting. Moving forward, perioperative spatial profiling, integrated imaging–liquid biopsy monitoring, and truly multidisciplinary workflows will be essential to translate surgical biology into actionable postoperative strategies.

## Data Availability

No new data were created or analyzed in this study. Data sharing is not applicable to this article. Figures were generated using ChatGPT 5.3 (OpenAI, San Francisco, CA, USA) and further refined using BioRender (BioRender.com, Toronto, ON, Canada), Adobe Photoshop (Adobe Inc., San Jose, CA, USA), and Microsoft PowerPoint (Microsoft Corporation, Redmond, WA, USA).
